# *E*-Cyanoacrylamides and 5-Imino Pyrrolones against *Trypanosoma cruzi*: Activity and Induced Mechanisms of Cell Death

**DOI:** 10.3390/tropicalmed9090191

**Published:** 2024-08-24

**Authors:** Carlos J. Bethencourt-Estrella, Samuel Delgado-Hernández, Atteneri López-Arencibia, Irene Serafín-Pérez, Paula Rodríguez-Santana, Sara Rodríguez-Camacho, Carolina Fernández-Serafín, David Tejedor, Jacob Lorenzo-Morales, José E. Piñero

**Affiliations:** 1 Instituto Universitario de Enfermedades Tropicales y Salud Pública de Canarias, Universidad de La Laguna, Avda. Astrofísico Fco. Sánchez, S/N, 38203 La Laguna, Tenerife, Islas Canarias, Spain; cbethene@ull.edu.es (C.J.B.-E.); atlopez@ull.edu.es (A.L.-A.); ireneser@ull.edu.es (I.S.-P.); alu0101254340@ull.edu.es (P.R.-S.); sararodcam@funccet.org (S.R.-C.); cfserafin@ull.edu.es (C.F.-S.); 2 Departamento de Obstetricia y Ginecología, Pediatría, Medicina Preventiva y Salud Pública, Toxicología, Medicina Legal y Forense y Parasitología, Universidad de La Laguna, 38203 La Laguna, Tenerife, Islas Canarias, Spain; 3 Centro de Investigación Biomédica en Red (CIBER) de Enfermedades Infecciosas (CIBERINFEC), Instituto de Salud Carlos III, 28220 Madrid, Spain; 4 Instituto de Productos Naturales y Agrobiología, Consejo Superior de Investigaciones Científicas, Avda. Fco. Sánchez 3, 38206 La Laguna, Tenerife, Islas Canarias, Spain; sdelgadh@ull.edu.es

**Keywords:** *Trypanosoma cruzi*, cyanoacrilamides, iminopirrolones, chemoterapy, Chagas, programmed cell death, ADME

## Abstract

Chagas disease is caused by a protozoan parasite called *Trypanosoma cruzi*. The infection produces a first clinical phase, commonly asymptomatic or showing non-specific symptoms, and a second chronic phase characterized by cardiac and digestive dysfunctions in some individuals with the disease. This disease affects 7 million people and has been categorized by the World Health Organisation as a neglected tropical disease. In addition, the drugs used to combat it were developed in the 1970s and present major toxicity problems and limited efficacy in the chronicity of the disease. This has led to research into new active compounds that are effective against the disease, with studies on cyanoderivatives showing promising activity. In this work, eight active *E*-cyanoacrylamides/5-imino pyrrolones were studied. Compounds B and F showed excellent activity, while compounds C and G stood out for their lower cytotoxicity. After correlating the activity and cytotoxicity of the compounds, it was observed that compounds B, C, and G obtained the most favourable results. Various cell death studies were carried out with these compounds, and it was determined that all of them produced programmed cell death, with compound B standing out as being at a late stage in the process.

## 1. Introduction

*Trypanosoma cruzi* is a protozoan parasite belonging to the Trypanosomatidae family that causes Chagas disease, discovered by Carlos Chagas in 1909, although previously described by Charles Darwin after his contact with the vector [1]. The World Health Organization considers this disease to be one of the twenty neglected tropical diseases [2] spreading mainly in Latin America, where the three main triatomine or bedbug vectors of this parasite are found: *Triatoma infestans*, *Rhodnius prolixus*, and *Triatoma dimidiata* [1].

Although *T. cruzi* infection occurs mainly through contact with the vector, with increasing infections through the ingestion of triatomine feces in contaminated food, it can also happen in the absence of the vector through blood transfusions, transplants, and transplacental transmission [3].

Chagas disease has mainly two clinical phases. The acute phase of the disease begins 1–2 weeks after infection and lasts approximately 8 weeks. At this point of infection, parasitemia is high, but most patients remain asymptomatic or have non-specific symptoms such as fever and malaise. In addition, chagomas of inoculation may appear at the bite site or, if the route of infection is the conjunctiva, a Romaña sign [4]. The patients will pass into the asymptomatic indeterminate phase after eight weeks. Throughout 10 to 30 years, 30% of the patients will develop organ dysfunction, mainly in cardiac tissue, leading to chagas cardiomyopathy, and in digestive tissue, characterized by enlarged viscera [4,5]; this is known as the chronic phase.

The World Health Organization (WHO) estimates that around 7 million people have Chagas disease, mainly in 21 Latin American countries that are recognized as endemic areas [6]. In these areas, Chagas disease is the parasitic disease with the greatest socio-economic impact, causing around 7500 deaths per year and more than 800,000 disability-adjusted years of life [7]. In addition, migratory movements have led to an increase in infection in non-endemic areas such as the United States and Europe, among others (Pinazo and Gascon, 2015 [8]).

Despite the importance of this disease, the treatments for this pathology are the same as in the 1970s: benznidazole and nifurtimox [9]. These nitroheterocyclics are prodrugs activated within the parasite by an enzyme [9] and act by directly inhibiting parasite DNA synthesis or by indirect interactions of metabolites of the nitro reduction process with parasite components [10]. These drugs are mainly effective against the acute phase of the disease, as well as in cases of chronic phase reactivation, women with the disease who are of childbearing age, and chronic patients up to 18 years of age [7]. However, the efficacy of these drugs decreases with increasing age and chronicity of the disease, as well as presenting multiple adverse effects such as paresthesia and gastrointestinal symptoms [11].

In recent years, research has focused on finding new drugs that are effective and have a lower adverse effect profile. Compounds such as fexinidazole [12] and other nitroderivatives [13], with similar groups to the reference molecules, and cyanoderivatives such as various acrylonitriles [14], which are effective against cruzipain, an enzyme essential for parasite survival [15,16], are among the investigated drugs.

Following this line of study, the present study analyzed the activity and cytotoxicity of different cyanoderivative compounds and the type of cell death they produce.

## 2. Materials and Methods

### 2.1. Cultures

Epimastigotes of *Trypanosoma cruzi* strain Y, provided by the Instituto de Parasitología y Biomedicina “López-Neyra” (IPBLN) belonging to the Consejo Superior de Investigaciones Científicas (CSIC) in Granada, Spain, were cultured in LIT medium and maintained at 26 °C.

Murine macrophages J774.A1 strain ATCC #TIB-67, acquired from American Type Culture Collection (ATTC, LGC Promochem, Barcelona, Spain), were cultured in DMEM medium and maintained at 37 °C and 5% CO_2_.

The L3 larvae from *Culex pipiens*, used in the cytotoxicity assay, were cultured at the Laboratorio de Entomología Médica of the Instituto Universitario de Enfermedades Tropicales y Salud Pública de Canarias in Tenerife, Spain.

### 2.2. Compounds

The eight tested compounds (Table 1) were synthesized, according to the method previously described [17], in the Instituto de Productos Naturales y Agrobiología of Consejo Superior de Investigaciones Científicas, La Laguna, Spain. The compounds were provided in powder form and subsequently dissolved in dimethyl sulfoxide (DMSO) and stored at 4 °C.

**Figure 1:**
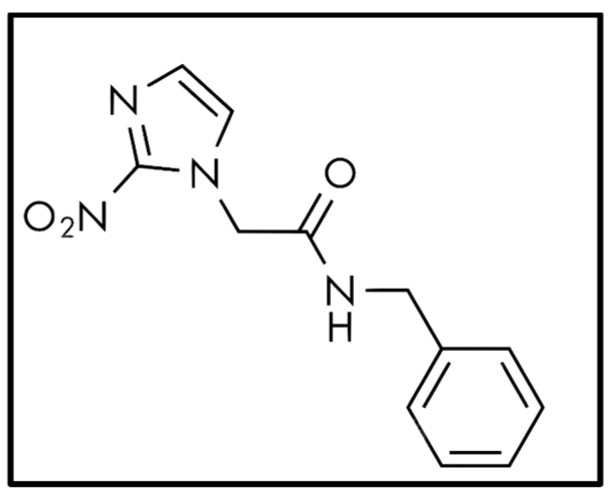
Molecular structure of benznidazole used as reference treatment.

Benznidazole (Figure 1), acquired from Sigma-Aldrich, Merck, was dissolved and preserved in the same way.

### 2.3. In Vitro Epimastigote Activity Assay

This assay is based on the use of alamarBlue. The alamarBlue reagent contains resazurin, which emits blue fluorescence under normal conditions. However, when the parasites are still alive and in contact with cell metabolism, the reagent is reduced to resorufin, which emits pink fluorescence.

In 96-well plates, serial dilutions of each compound were added with parasites at a concentration of 10^6^ cells/mL in a final volume of 200 µL. Finally, 10% alamarBlue reagent was added, with 20 µL per well.

After 72 h of incubation at 26 °C, the plate was introduced into the plate reader and the fluorescence (570 nm excitation, 585 nm emission) of each well was determined using the EnSpire Multimode Plate Reader^®^ (PerkinElmer, Thermo Fischer Scientific, Madrid, Spain). Finally, using a non-linear regression analysis in Graphpad Prism 9.0.0. the inhibitory concentration 50 (IC_50_) was calculated.

### 2.4. In Vitro Amastigote Activity Assay

To perform the assay against the intracellular form of the parasite, the same colorimetric method based on the alamarBlue was developed. To produce the internalization of the parasites, an excess of epimastigote forms were added (10^5^:10^4^ parasites/macrophages in 100 µL of DMEM) to a 96-well plate for 24 h at 37 °C. Then, the wells were washed 3 times to eliminate the non-internalized parasites, and the serial dilutions of the compounds were added and dissolved in DMEM medium. After another incubation of 24 h at 37 °C, the drugs were eliminated and the cells were lysed with 0.05% SDS for 30 s. After adding LIT in a final volume of 200 µL and 10% of alamarBlue™ reagent, the plates were incubated for 96 h to promote the externalization of the parasites. Finally, the fluorescence was read using the EnSpire Multimode Plate Reader^®^ and the inhibitory concentration 50 (IC_50_) was calculated.

### 2.5. Cytotoxicity and Larvicidal Assay

This assay followed the same principle as the previous one, but in this case, macrophages were used instead of parasites to determine the toxicity of the compounds. A total of 50 μL with a concentration of 2 × 10^5^ cells/mL was added to each well with serial dilutions of the compounds in 50 μL of RPMI medium. After this, 10% of alamarBlue™ reagent was added and the plate was incubated at 37 °C and 5% CO_2_ for 24 h. After this time, the fluorescence was read using the EnSpire Multimode Plate Reader^®^ and the cytotoxic concentration 50 (IC_50_) was calculated.

In the most selective compounds, an in vivo test against mosquito larvae was also carried out to confirm the low toxicity of the compounds. For this purpose, 3 mL of sterile water with food for 48 h was added to 24-well plates, following WHO guidelines of necessary nutrients [18]. In each well, one L3 larva of *Culex pipiens* was added, together with different concentrations of the compounds to be tested (IC_90_ and two times IC_90_). In addition, negative controls, without treatment and with the maximum amount of DMSO added in the treatments, and positive controls, benznidazole as the reference treatment and triton, previously reported to be toxic to larvae, were added. All experiments were performed in duplicate and repeated on 3 different days, observing larval viability at different times (1, 3, 12, 24, and 48 h after the start of treatment) [19].

### 2.6. Induction of Cell Death Mechanisms 

The process of programmed cell death (PCD) is characterized by the presence of certain events that can be demonstrated using different commercial kits. To observe these events, parasites were incubated for 24 h with the IC_90_ of the compounds. After that, the plate was centrifuged at 3000 rpm at 4 °C for 10 min to remove the supernatant. The pellet was then resuspended in buffer and the different kits were added following the manufacturer’s instructions.

#### 2.6.1. Assessment of Cellular ATP Level

ATP is an essential molecule for cells to carry out vital processes. Therefore, a decrease in the ATP levels could lead to instability of these functions, which are essential for the survival of the cell, and would therefore be related to a possible PCD process.

CellTiter-Glo^®^ Luminescent Cell Viability Assay was used to determine the ATP levels of the parasitic cells. This kit contains luciferin upon contact with cell metabolism, oxidises to oxyluciferin and emitting luminescence.

The procedure was as follows: after 24 h of incubation and centrifugation, the pellet was resuspended in 25 μL of buffer and mixed with 25 μL of CellTiter-Glo^®^ in the dark, shaking for 2 min. Then, after 15 min of incubation at room temperature, the luminescence was read in the EnSpire Multimode Plate Reader^®^ [20].

#### 2.6.2. Determination of the Integrity of the Plasma Membrane

The plasma membrane is the first cellular defense against external agents and plays a fundamental role in substance exchange processes. Thus, any disruption in the membrane is a critical point in cell survival.

In PCD, there is an alteration in the permeability of the plasma membrane, but the cell structure is maintained. The SYTOX^™^ Green Stain (Thermo Fischer Scientific, Madrid, Spain) only penetrates cells with increased plasma permeability. Once inside the cell, it will bind to the DNA and emit green fluorescence.

After incubation and centrifugation, the pellet was resuspended in 50 μL of buffer and the reagent was added at a concentration of 1 μM. After 30 min of waiting, the fluorescence was observed on the EVOS microscope in the GFP channel, which also allows imaging [21].

#### 2.6.3. Determination of Mitochondrial Membrane Alterations

The mitochondria are essential for cell survival, performing vital functions in metabolism and energy production. A JC-1 Mitochondrial Membrane Potential Assay Kit^®^ (Cayman Chemical, Ann Arbor, MI, USA) was used to determine the levels of mitochondrial membrane potential of the parasitic cells.

After 24 h of incubation and centrifugation, the pellet was resuspended in 50 μL of buffer with 5 µL of the kit, in the dark. Then, after 30 min of incubation at room temperature, the fluorescence was read in the EnSpire Multimode Plate Reader^®^. The results were expressed as the J-monomers (green fluorescence, excitation 540 nm/emission 470 nm)-to-J-aggregates (red fluorescence, excitation 485 nm/emission 535 nm) ratio [22].

#### 2.6.4. Chromatin Condensation Analysis

One of the most revealing events of PCD is the presence of chromatin condensation. To assess the occurrence of this event, the Vybrant™ Apoptosis Assay Kit (Thermo Fischer Scientific, Madrid, Spain) was used, which features two reagents: Hoechst, a dye that penetrates intact cells, binding to condensed DNA and emitting blue fluorescence, and propidium iodide (PI), an intercalating agent that only penetrates death cells, which binds to DNA and emits red fluorescence.

The pellet was resuspended in 50 μL of buffer with the reagents, 5 µg/mL of Hoechst, and 1 μg/mL of PI. After 30 min of incubation, fluorescence was observed under the EVOS microscope in DAPI and RFP channels, and the images were captured [22].

#### 2.6.5. Determination of the Presence of Reactive Oxygen Species

Cellular metabolism elevates the production of reactive oxygen species (ROS) that are eliminated by cellular mechanisms. However, when the cell enters a PCD process, it is unable to eliminate these substances, which accumulate, producing oxidative stress. To measure the presence of ROS, CellROX™ Deep Red was used. This kit contains a reagent capable of penetrating cells, which in the presence of ROS oxidizes and emits red fluorescence.

The pellet obtained after centrifugation was resuspended in 50 μL of buffer and the reagent was added at a concentration of 5 μM. After incubation for 30 min at room temperature, the fluorescence was observed under the EVOS microscope with the Cy5 channel, and the images were captured [23].

### 2.7. ADME-Related Calculation

To predict the ADME-related calculation of the tested compounds, the SwissADME web tool (Swiss Institute of Bioinformatics, Lausanne, Switzerland) was used. This tool has access to predictive models for physicochemical properties, pharmacokinetics, drug likeness, and medicinal chemistry friendliness [24].

### 2.8. Statistical Analyses

Inhibitory and cytotoxic concentrations (IC_50_, IC_90_, and CC_50_) were determined in duplicate on three different days using non-linear regression [Inhibitor] vs. response variable slope (four parameters) using non-linear regression analysis with 95% confidence, using GraphPad Prism 10.1.1 statistical software. The analysis was developed using Tukey’s test, considering significant values of *p* < 0.05. ANOVA analysis was also performed using GraphPad Prism 10.1.1.

## 3. Results

### 3.1. In Vitro Inhibition of Epimastigote Activity

The results of inhibitory concentration 50 (IC_50_) against the epimastigote form of *T. cruzi* are included in Table 2, where most of the compounds showed an activity lower than 100 µM.

### 3.2. In Vitro Anti-Mmastigote Activity

In the case of intracellular parasites, the activity, presented as IC_50_ against amastigote forms, are included in Table 3. In this case, only the most selective compounds against epimastigote forms (B, C, and G) were included to perform the intracellular activity.

### 3.3. Cytotoxicity of the Tested Compounds

The results of cytotoxic concentration 50 (CC_50_) against murine macrophages are included in Table 4.

The relation between the activity and cytotoxicity, to determine the selectivity of the compounds, was calculated as a ratio (CC_50_/IC_50_). The results are included in Table 5 for the epimastigote form and in Table 6 for the intracellular form.

To ensure low toxicity of the compounds, the results of toxicity against L3 larvae of *Culex pipiens* are included in Table 7. The larvae were checked at different moments, from 1 h to 48 h of incubation, dividing between live and dead larvae.

### 3.4. Observed Induction of Mechanisms of Cell Death

#### 3.4.1. Cellular ATP Levels Monitoring

**Figure 2:**
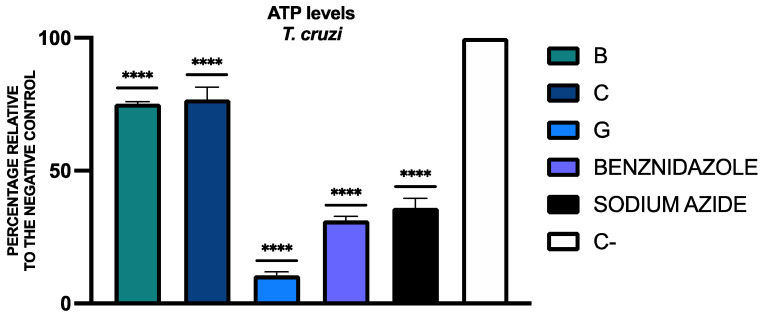
Results of ATP levels presented as a percentage relative to the negative control (C-, without any treatment). A Tukey test with GraphPad.PRISM^®^ 10.1.1 software was conducted to test the statistical differences between means (*p* < 0.0001 [****]). Benznidazole was added as the reference treatment and sodium azide was used as the positive control.

The results of ATP levels are included in Figure 2. The results of the tested compounds are expressed as a percentage relative to the negative control (C-), considering the luminescence of the negative control without any treatment as 100%.

#### 3.4.2. Permeability of Plasmastic Membrane

**Figure 3:**
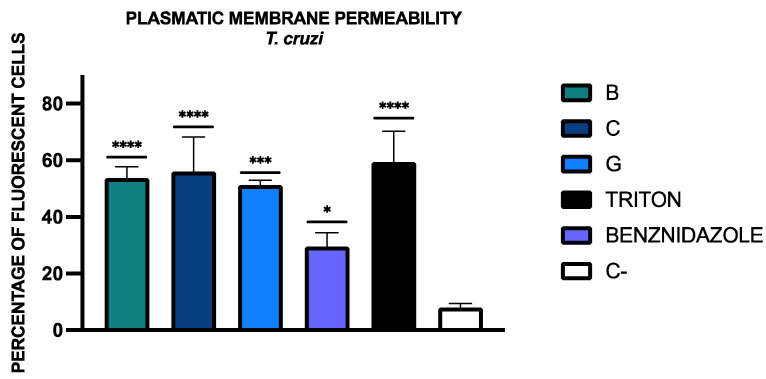
Results of plasmatic membrane permeability levels presented as a percentage of fluorescent cells, and the statistical analysis was conducted relative to the negative control (C-, without treatment). A Tukey test with GraphPad.PRISM^®^ 10.1.1 software was conducted to test the statistical differences between means (*p* < 0.05 [*]; *p* < 0.001 [***]; *p* < 0.0001 [****]). Benznidazole was added as the reference treatment and triton was used as the positive control.

To determine the plasmatic membrane permeability, the percentage of fluorescent cells was calculated and is included in Figure 3. The statistical analysis of the results was performed relative to the negative control (C-). Fluorescence images could be observed in the Figure S2.

#### 3.4.3. Mitochondrial Membrane Potential Alterations

**Figure 4:**
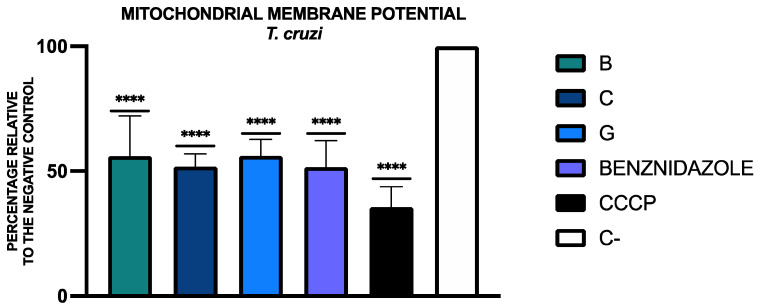
Results of mitochondrial membrane potential alterations, expressed as a percentage relative to the negative control (C-, without any treatment). A Tukey test with GraphPad.PRISM^®^ 10.1.1 software was conducted to test the statistical differences between means. (*p* < 0.0001 [****]). Benznidazole was added as the reference treatment and carbonyl cyanide m-chlorophenyl hydrazone (CCCP) was used as the positive control.

The ratio of red/green fluorescence was expressed as a percentage relative to the negative control (C-), considering the ratio of the negative control without any treatment as 100%. The results and the statistical analysis are included in Figure 4.

#### 3.4.4. Assesment of Chromatin Condensation 

**Figure 5:**
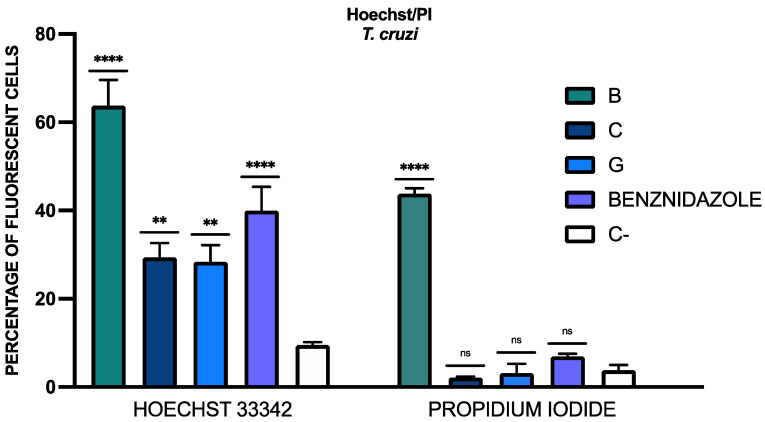
Results of double stain Hoechst/PI, expressed as a percentage relative to the negative control (C-, without any treatment). A Tukey test with GraphPad.PRISM^®^ 10.1.1 software was conducted to test the statistical differences between means. (*p* > 0.05, non-significative [^ns^]; *p* < 0.01 [**]; *p* < 0.0001 [****]). Benznidazole was added as the reference treatment.

To see the presence of chromatin condensation, the double stain Vybrant™ Apoptosis Assay Kit was used. The fluorescent cells with Hoechst 33342 were those which exhibited chromatin condensation in the DAPI channel and the fluorescent cells with propidium iodide were those that were dead. The results of the percentage of fluorescent cells is included in Figure 5. The statistical analysis was performed relative to the negative control (C-), without any treatment. Fluorescence images could be observed in the Figure S1.

#### 3.4.5. Generation of Reactive Oxygen Species

**Figure 6:**
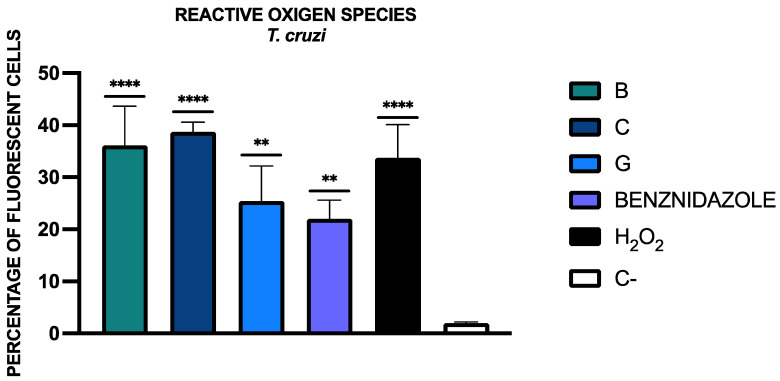
Results of the accumulation of reactive oxygen species (ROS), expressed as a percentage relative to the negative control (C-, without any treatment). A Tukey test with GraphPad.PRISM^®^ 10.1.1 software was conducted to test the statistical differences between means. (*p* < 0.01 [**]; *p* < 0.0001 [****]). Benznidazole was added as the reference treatment and oxygen peroxide as the positive control.

Using the CellROX reagent, the cells that have ROS accumulation emit fluorescence in the Cy5 channel. The percentage of fluorescent cells in the Cy5 channel is represented in Figure 6. The statistical analysis was performed relative to the negative control (C-), without treatment. Fluorescence images could be observed in the Figure S3.

### 3.5. Prediction of ADME Parameters and Pharmacokinetic Properties

**Figure 7:**
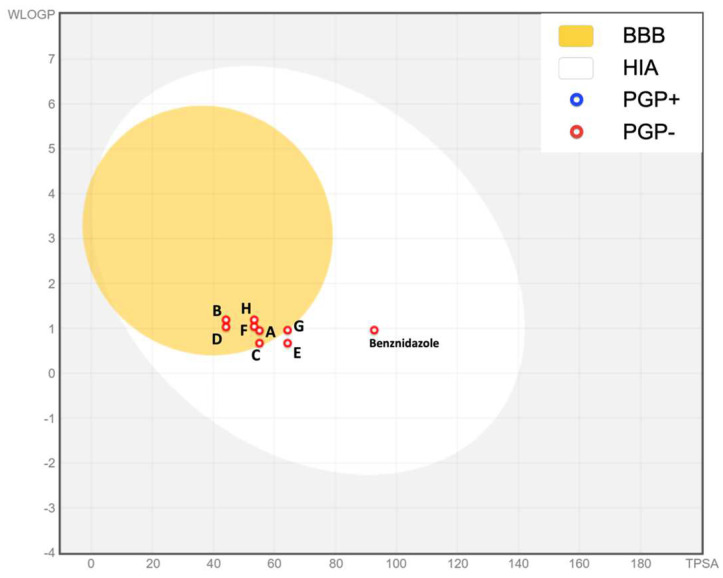
Boiled-egg graph representing the SwissADME predictions. The yellow area refers to the probability to pass the blood–brain barrier and the white area represents the high probability of absorption in the gastrointestinal tract. Each compound is presented as a point; those that were not shown to be P-gp substrates (PGP-) are indicated in red.

The results of the prediction of ADME parameters and pharmacokinetic properties are represented in Figure 7. The results demonstrate that some compounds, including those in the yellow area, (A, B, C, D, F, and H) would pass the blood–brain barrier (BBB). On the other hand, all the compounds, including the reference treatment benznidazole, would be absorbed in the gastrointestinal tract (HIA). In addition, all of them are not substrates of P-gp (PGP-), so they would not bind to it.

Another predictable point with the SwissADME analysis is the possibility of oral bioavailability. This prediction consists of the study of different properties based on pre-established rules such as Lipinski (Pfizer), Ghose, Veber (GSK), Egan (Pharmacia), and Muegge (Bayer). The results show that all compounds appear to be good candidates for oral absorption, and almost all the rules studied are fulfilled, as shown in Table 8.

The SwissADME also allows the prediction of the behavior of drugs against cytochrome P450. A large part of drug excretion is carried out by the hepatic route via cytochrome P450 enzymes, so the inhibition of these enzymes could lead to a decrease in excretion and the consequent accumulation and toxicity of the drug. The results of the inhibition of the most important CYP human enzymes, involved in the excretion of 90% of drugs (1A2, 2C19, 2C9, 2D6, and 3A4), are shown in Table 9. The compounds that produce an inhibition in the CYP enzymes are marked with an X.

## 4. Discussion

All the tested compounds, except compound E, showed activity under 100 µM against epimastigote forms of *T. cruzi*, with compounds B, F, and H standing out for their excellent activity against the parasite (IC_50_ < 10 μM). Likewise, observing their toxicity on murine macrophages, compounds C, E, and G stood out for their cytotoxicity (CC_50_ > 200 μM).

Once their selectivity index was determined against the epimastigote forms of the parasite, it was observed that the compounds with the most favorable ratio were B, C, and G, since their SI values were above 10. Thus, these compounds were selected to determine the activity against the intracellular forms of the parasite and to evaluate the induction of programmed cell death.

Concerning the structure activity of *E*-cyanoacrylamides/5-imino pyrrolones, it was clearly observed that in each isomer pair, the trans- or *E*-isomers (A, C, E, and G) showed a lower activity than the cis- or *Z*-isomers (B, D, F, and H). In addition, there was a notable difference in toxicity, where the *E* isomers appeared to be less toxic than the *Z* isomers, the pair of G–H isomers standing out, and the *Z* isomer being 100 times more toxic than the *E* isomer.

Analysis of the different mechanisms reveals the presence of different events related to apoptotic or programmed cell death [25,26]. It is plausible to conclude that all compounds produced a programmed cell death process, highlighting compound B, where the parasites were in a late phase of this type of cell death. This conclusion was reached by analyzing all the mechanisms together, differentiating the late programmed cell death process by the presence of fluorescence with a propidium iodide reagent.

The compounds studied appear to have a good bioavailability according to the ADME predictions. Good intestinal absorption and the absence of P-gp binding make these drugs good candidates for use. In addition, compliance with almost all standards for a drug to be suitable for oral administration makes these compounds candidates for oral administration [27]. On the other hand, the impossibility of passing through the blood–brain barrier and the lack of inhibition of CYP enzymes, could mean that these drugs will be less likely to have problems related to neurotoxicity due to entry into the brain compartment or cumulative toxicity due to lack of excretion by CYP 450 [28].

In addition, this study again demonstrates the trypanocidal activity of the cyanoderivative compounds mentioned above. This confirms previous studies, where other cyanoderivative compounds appeared to be good candidates for the treatment of Chagas disease, such as cyanomethyl vinyl ethers [29], selenocyanate derivatives [30], or Heterocyclic-2-carboxylic acid (3-Cyano-1,4-di-N-oxidequinoxalin-2-yl) amide derivatives [31]. Therefore, cyanoderivative compounds constitute a potential line of research for the treatment of *Trypanosoma cruzi* infection.

## 5. Conclusions

In conclusion, the cyanoderivative compounds showed activity against *Trypanosoma cruzi* by producing programmed cell death. These results confirmed previous studies indicating that compounds with cyano groups are effective against this protozoan. The cyanoderivatives are proposed as candidates for further studies and possible use as a future antichagasic agent.

## Figures and Tables

**Table 1:** Molecular structure of tested compounds. *E*-cyanoacrylamides (A, C, E, and G) and 5-imino pyrrolones (B, D, F and H).

*E*-Cyanoacrylamides		5-Imino Pyrrolones	
**A**	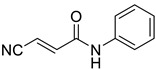	B	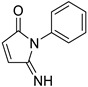
**C**	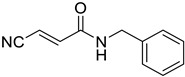	D	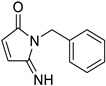
**E**	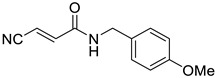	F	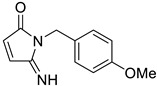
**G**	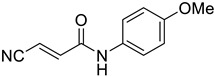	H	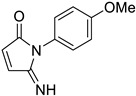

**Table 2:** Results of activity against the epimastigote form of *T. cruzi*. All the results are expressed as mean ± standard deviation in µM. Benznidazole was added as the reference treatment.

Compound	IC_50_	Compound	IC_50_
**A**	18.68 ± 1.90	E	>100
**B**	6.91 ± 1.33	F	3.47 ± 0.58
**C**	18.78 ± 2.03	G	20.72 ± 2.66
**D**	12.36 ± 1.49	H	2.58 ± 0.52
		Benznidazole	6.92 ± 0.77

**Table 3:** Results of activity against the amastigote form of *T. cruzi*. The results are expressed as mean ± standard deviation in µM. Benznidazole was added as the reference treatment.

**Compound**	**IC_50_**	**Compound**	**IC_50_**
**B**	10.78 ± 2.11	G	10.59 ± 3.05
**C**	24.14 ± 6.22	Benznidazole	2.67 ± 0.39

**Compound**

**IC_50_**

**Compound**

**IC_50_**

**Table 4:** Results of cytotoxicity against the murine macrophages. All the results are expressed in mean ± standard deviation, in µM. Benznidazole was added as the reference treatment.

Compound	IC_50_	Compound	IC_50_
**A**	94.78 ± 23.11	E	319.61 ± 60.22
**B**	118.07 ± 11.90	F	2.82 ± 1.39
**C**	208.01 ± 13.44	G	266.62 ± 42.48
**D**	85.32 ± 3.71	H	2.42 ± 1.58
		Benznidazole	399.91 ± 1.40

**Table 5:** Results of selectivity indexes against the epimastigote form of *T. cruzi*. Benznidazole was added as the reference treatment.

Compound	SI	Compound	SI
**A**	5.1	E	<3.2
**B**	17.1	F	0.8
**C**	11.1	G	12.9
**D**	6.9	H	0.9
		Benznidazole	57.8

**Table 6:** Results of selectivity indexes against the amastigote form of *T. cruzi*. Benznidazole was added as the reference treatment.

Compound	SI	Compound	SI
**B**	10.9	G	25.2
**C**	8.6	Benznidazole	149.8

**Table 7:** Results of toxicity against L3 larvae of *Culex pipiens*. Benznidazole (Benz) was added as the reference treatment and triton was added as positive control. As a negative control, some L3 was added only in water (C-) and with only DMSO.

Compound	1 h	3 h	12 h	24 h	48 h
IC_90_ B	Alive	Alive	Alive	Alive	Alive
2IC_90_ B	Alive	Alive	Alive	Alive	Alive
IC_90_ C	Alive	Alive	Alive	Alive	Alive
2IC_90_ C	Alive	Alive	Alive	Alive	Alive
IC_90_ G	Alive	Alive	Alive	Alive	Alive
2IC_90_ G	Alive	Alive	Alive	Alive	Alive
IC_90_ Benz	Alive	Alive	Alive	Alive	Alive
2IC_90_ Benz	Alive	Alive	Alive	Alive	Alive
Triton 0.01%	Alive	Alive	Dead	Dead	Dead
Triton 0.5%	Alive	Dead	Dead	Dead	Dead
Triton 1%	Dead	Dead	Dead	Dead	Dead
DMSO 12 µL	Alive	Alive	Alive	Alive	Alive
C-	Alive	Alive	Alive	Alive	Alive

**Table 8:** Results of druglikeness based on five different rules, including Lipinski (Pfizer), Ghose, Veber (GSK), Egan (Pharmacia), and Muegge (Bayer). Rules that are not complied with are marked with an X.

	Druglikeness				
	Lipinski	Ghose	Veber	Egan	Muegge
A					X
B					X
C					X
D					X
E					
F					
G					
H					
Benznidazole					

**Table 9:** Pharmacokinetics based on the prediction of the inhibition of the cytochrome P450 (CYP).

	CYP Inhibitors				
	1A2	2C19	2C9	2D6	3A4
A					
B					
C					
D					
E					
F	X				
G					
H	X				
Benznidazole					

## Data Availability

Any data are available upon request from the authors.

## Supplementary Materials

The following supporting information can be downloaded at: https://www.mdpi.com/article/10.3390/tropicalmed9090191/s1, Figure S1: Results of detection of chromatin condensation using Hoechst/PI; Figure S2: Results of detection of plasmatic membrane permeability in epimastigote stage of *T. cruzi* using SYTOX^®^ Green staining; Figure S3: Results of detection of reactive oxygen species in epimastigote forms of *T. cruzi* using CellROX^®^ Deep Red staining.

## References

[1] Guarner J. (2019). Chagas disease as example of a reemerging parasite. Semin. Diagn. Pathol..

[2] World Health Organization (WHO) (2010). First WHO Report on Neglected Tropical Diseases: Working to Overcome the Global Impact of Neglected Tropical Diseases.

[3] Santos É., Menezes Falcão L. (2020). Chagas cardiomyopathy and heart failure: From epidemiology to treatment. Rev. Port. Cardiol..

[4] Bern C., Messenger L.A., Whitman J.D., Maguire J.H. (2019). Chagas Disease in the United States: A Public Health Approach. Clin. Microbiol. Rev..

[5] Rodrigues J.C.F., Godinho J.L.P., de Souza W. (2014). Biology of human pathogenic trypanosomatids: Epidemiology, lifecycle and ultrastructure. Subcell. Biochem..

[6] WHO (2017). Integrating Neglected Tropical Diseases into Global Health and Development: Fourth WHO Report on Neglected Tropical Diseases.

[7] Kratz J.M. (2019). Drug discovery for chagas disease: A viewpoint. Acta Trop..

[8] Pinazo M.J., Gascon J. (2015). The importance of the multidisciplinary approach to deal with the new epidemiological scenario of Chagas disease (global health). Acta Trop..

[9] García-Huertas P., Cardona-Castro N. (2021). Advances in the treatment of Chagas disease: Promising new drugs, plants and targets. Biomed. Pharmacother..

[10] Chao C., Leone J.L., Vigliano C.A. (2020). Chagas disease: Historic perspective. Biochim. Biophys. Acta. Mol. Basis Dis..

[11] Sales Junior P.A., Molina I., Fonseca Murta S.M., Sánchez-Montalvá A., Salvador F., Corrêa-Oliveira R., Carneiro C.M. (2017). Experimental and Clinical Treatment of Chagas Disease: A Review. Am. J. Trop. Med. Hyg..

[12] Mathias F., Kabri Y., Brun D., Primas N., Di Giorgio C., Vanelle P. (2022). Synthesis and Anti-Trypanosoma cruzi Biological Evaluation of Novel 2-Nitropyrrole Derivatives. Molecules.

[13] Palace-Berl F., Jorge S.D., Pasqualoto K.F.M., Ferreira A.K., Maria D.A., Zorzi R.R., de Sá Bortolozzo L., Lindoso J.Â.L., Tavares L.C. (2013). 5-Nitro-2-furfuriliden derivatives as potential anti-*Trypanosoma cruzi* agents: Design, synthesis, bioactivity evaluation, cytotoxicity and exploratory data analysis. Bioorg. Med. Chem..

[14] Bethencourt-Estrella C.J., Delgado-Hernández S., López-Arencibia A., San Nicolás-Hernández D., Sifaoui I., Tejedor D., García-Tellado F., Lorenzo-Morales J., Piñero J.E. (2021). Acrylonitrile Derivatives against *Trypanosoma cruzi*: In Vitro Activity and Programmed Cell Death Study. Pharmaceuticals.

[15] Avelar L.A.A., Camilo C.D., de Albuquerque S., Fernandes W.B., Gonçalez C., Kenny P.W., Leitão A., McKerrow J.H., Montanari C.A., Orozco E.V.M. (2015). Molecular Design, Synthesis and Trypanocidal Activity of Dipeptidyl Nitriles as Cruzain Inhibitors. PLoS Negl. Trop. Dis..

[16] Mott B.T., Ferreira R.S., Simeonov A., Jadhav A., Ang K.K.-H., Leister W., Shen M., Silveira J.T., Doyle P.S., Arkin M.R. (2010). Identification and optimization of inhibitors of Trypanosomal cysteine proteases: Cruzain, rhodesain, and TbCatB. J. Med. Chem..

[17] Chao-Pellicer J., Delgado-Hernández S., Arberas-Jiménez I., Sifaoui I., Tejedor D., García-Tellado F., Piñero J.E., Lorenzo-Morales J. (2024). Synthesis and Biological Evaluation of Cyanoacrylamides and 5-Iminopyrrol-2- ones Against Naegleria fowleri. ACS Infect. Dis..

[18] World Health Organization (WHO) (2005). Guidelines for Laboratory and Field Testing of Mosquito Larvicides.

[19] Bouabida H., Dris D. (2022). Biological toxicity of *Ruta graveolens* essential oil against three species of diptera *Drosophila melanogaster*, *Culex pipiens* and *Culiseta longiareolata*. J. Vector Borne Dis..

[20] San Nicolás-Hernández D., Hernández-Álvarez E., Bethencourt-Estrella C.J., López-Arencibia A., Sifaoui I., Bazzocchi I.L., Lorenzo-Morales J., Jiménez I.A., Piñero J.E. (2023). Multi-target withaferin-A analogues as promising anti-kinetoplastid agents through the programmed cell death. Biomed. Pharmacother..

[21] López-Arencibia A., San Nicolás-Hernández D., Bethencourt-Estrella C.J., Sifaoui I., Reyes-Batlle M., Rodríguez-Expósito R.L., Rizo-Liendo A., Lorenzo-Morales J., Bazzocchi I.L., Piñero J.E. (2019). Withanolides from *Withania aristata* as Antikinetoplastid Agents through Induction of Programmed Cell Death. Pathogens.

[22] Bethencourt-Estrella C.J., Delgado-Hernández S., López-Arencibia A., San Nicolás-Hernández D., Tejedor D., García-Tellado F., Lorenzo-Morales J., Piñero J.E. (2022). In vitro activity and cell death mechanism induced by acrylonitrile derivatives against *Leishmania amazonensis*. Bioorg. Chem..

[23] Bethencourt-Estrella C.J., Nocchi N., López-Arencibia A., San Nicolás-Hernández D., Souto M.L., Suárez-Gómez B., Díaz-Marrero A.R., Fernández J.J., Lorenzo-Morales J., Piñero J.E. (2021). Antikinetoplastid Activity of Sesquiterpenes Isolated from the Zoanthid *Palythoa aff*. clavata. Pharmaceuticals.

[24] Swiss Institute of Bioinformatics Swiss ADME Disponible en. http://www.swissadme.ch/.

[25] Ouaissi A. (2003). Apoptosis-like death in trypanosomatids: Search for putative pathways and genes involved. Kinetoplastid Biol. Dis..

[26] Smirlis D., Duszenko M., Ruiz A.J., Scoulica E., Bastien P., Fasel N., Soteriadou K. (2010). Targeting essential pathways in trypanosomatids gives insights into protozoan mechanisms of cell death. Parasites Vectors.

[27] Asano D., Takakusa H., Nakai D. (2023). Oral Absorption of Middle-to-Large Molecules and Its Improvement, with a Focus on New Modality Drugs. Pharmaceutics.

[28] Zhao M., Ma J., Li M., Zhang Y., Jiang B., Zhao X., Huai C., Shen L., Zhang N., He L. (2021). Cytochrome P450 Enzymes and Drug Metabolism in Humans. Int. J. Mol. Sci..

[29] Bethencourt-Estrella C.J., Delgado-Hernández S., López-Arencibia A., San Nicolás-Hernández D., Tejedor D., García-Tellado F., Lorenzo-Morales J., Piñero J.E. (2023). In vitro activity and mechanism of cell death induction of cyanomethyl vinyl ethers derivatives against *Trypanosoma cruzi*. Int. J. Parasitol. Drugs Drug Resist..

[30] Chao M.N., Lorenzo-Ocampo M.V., Szajnman S.H., Docampo R., Rodriguez J.B. (2019). Further insights of selenium-containing analogues of WC-9 against *Trypanosoma cruzi*. Bioorg. Med. Chem..

[31] Ancizu S., Moreno E., Torres E., Burguete A., Pérez-Silanes S., Benítez D., Villar R., Solano B., Marín A., Aldana I. (2009). Heterocyclic-2-carboxylic acid (3-cyano-1,4-di-N-oxidequinoxalin-2-yl)amide derivatives as hits for the development of neglected disease drugs. Molecules.

