# Supplementary material for: E-Cyanoacrylamides and 5-Imino Pyrrolones against Trypanosoma cruzi: Activity and Induced Mechanisms of Cell Death
Source: Trop Med Infect Dis. 2024 Aug 24;9(9):191. doi: 10.3390/tropicalmed9090191 (PMC11436024; doi:10.3390/tropicalmed9090191)
Supplement: Supplementary file 1 [file tropicalmed-09-00191-s001.zip › tropicalmed-3160513-supplementary.pdf]

Tropical Medicine and Infectious Disease

MDPI

Article

E-Cyanoacrylamides and 5-Imino Pyrrolones against *Trypanosoma cruzi*: Activity and Induced Mechanisms of Cell Death

Carlos J. Bethencourt-Estrella [1,2,3], Samuel Delgado-Hernández [4], Atteneri López-Arencibia [1,2,3], Irene Serafín-Pérez [1,2,3], Paula Rodríguez-Santana [1,2], Sara Rodríguez-Camacho [1,2], Carolina Fernández-Serafín [1,2,3], David Tejedor [4,*], Jacob Lorenzo-Morales [1,2,3,*] and José E. Piñero [1,2,3,*]

[1] Instituto Universitario de Enfermedades Tropicales y Salud Pública de Canarias, Universidad de La Laguna, Avda. Astrofísico Fco. Sánchez, S/N, 38203 La Laguna, Tenerife, Islas Canarias, Spain; cbethene@ull.edu.es (C.J.B.-E.); atlopez@ull.edu.es (A.L.-A.); ireneser@ull.edu.es (I.S.-P.); alu0101254340@ull.edu.es (P.R.-S.); sararodcam@funccet.org (S.R.-C.); cfserafin@ull.edu.es (C.F.-S.)
[2] Departamento de Obstetricia y Ginecología, Pediatría, Medicina Preventiva y Salud Pública, Toxicología, Medicina Legal y Forense y Parasitología, Universidad de La Laguna, 38203 La Laguna, Tenerife, Islas Canarias, Spain
[3] Centro de Investigación Biomédica en Red (CIBER) de Enfermedades Infecciosas (CIBERINFEC), Instituto de Salud Carlos III, 28220 Madrid, Spain
[4] Instituto de Productos Naturales y Agrobiología, Consejo Superior de Investigaciones Científicas, Avda. Fco. Sánchez 3, 38206 La Laguna, Tenerife, Islas Canarias, Spain; sdelgadh@ull.edu.es
* Correspondence: dtejedor@ipna.csic.es (D.T.); jmlorenz@ull.edu.es (J.L.-M.); jpinero@ull.edu.es (J.E.P.)

Supplementary Materials:

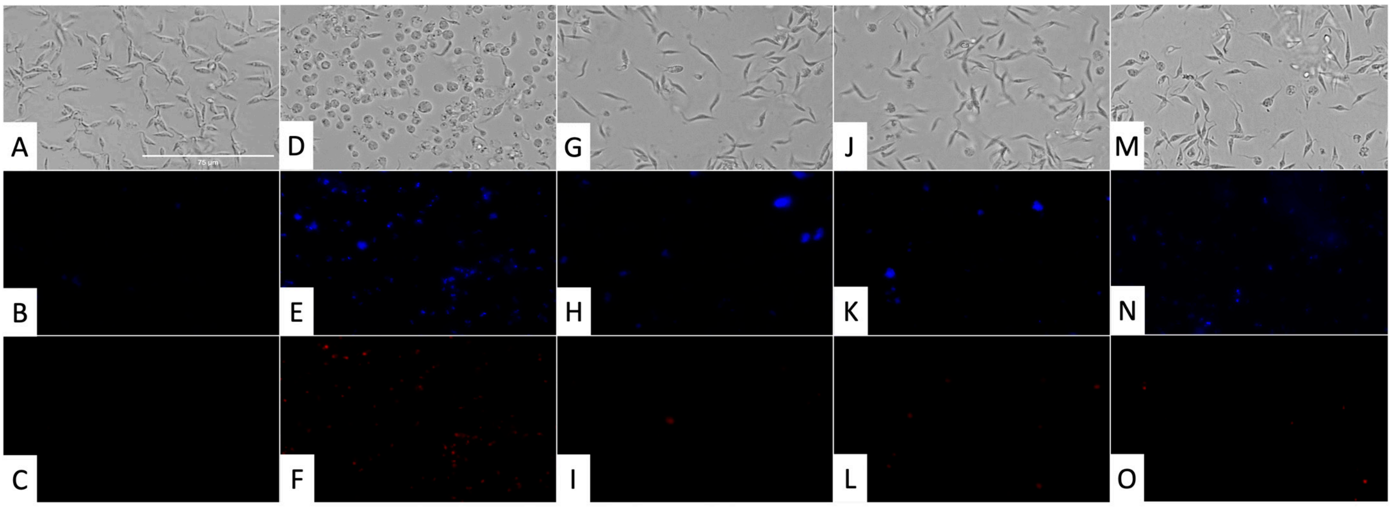

Figure S1. Results of detection of chromatin condensation using Hoechst/PI. Results after 24 hours of incubation with the IC_{90} against epimastigote forms of *T. cruzi*. Images were captured using an EVOS FL Cell Imaging System (40×). Benznidazole was used as reference treatment. A: Parasites without treatment in visible channel; B: Parasites without treatment in DAPI channel; C: Parasites without treatment in RFP channel; D: Parasites treated with B in visible channel; E: Parasites treated with B in DAPI channel; F: Parasites treated with B in RFP channel; G: Parasites treated with C in visible channel; H: Parasites treated with C in DAPI channel; I: Parasites treated with C in RFP channel; J: Parasites treated with G in visible channel; K: Parasites treated with G in DAPI channel; L: Parasites treated with G in RFP channel; M: Parasites treated with benznidazole in visible channel; N: Parasites treated with benznidazole in DAPI channel; O: Parasites treated with benznidazole in RFP channel. Scale-bar: 75 µm.

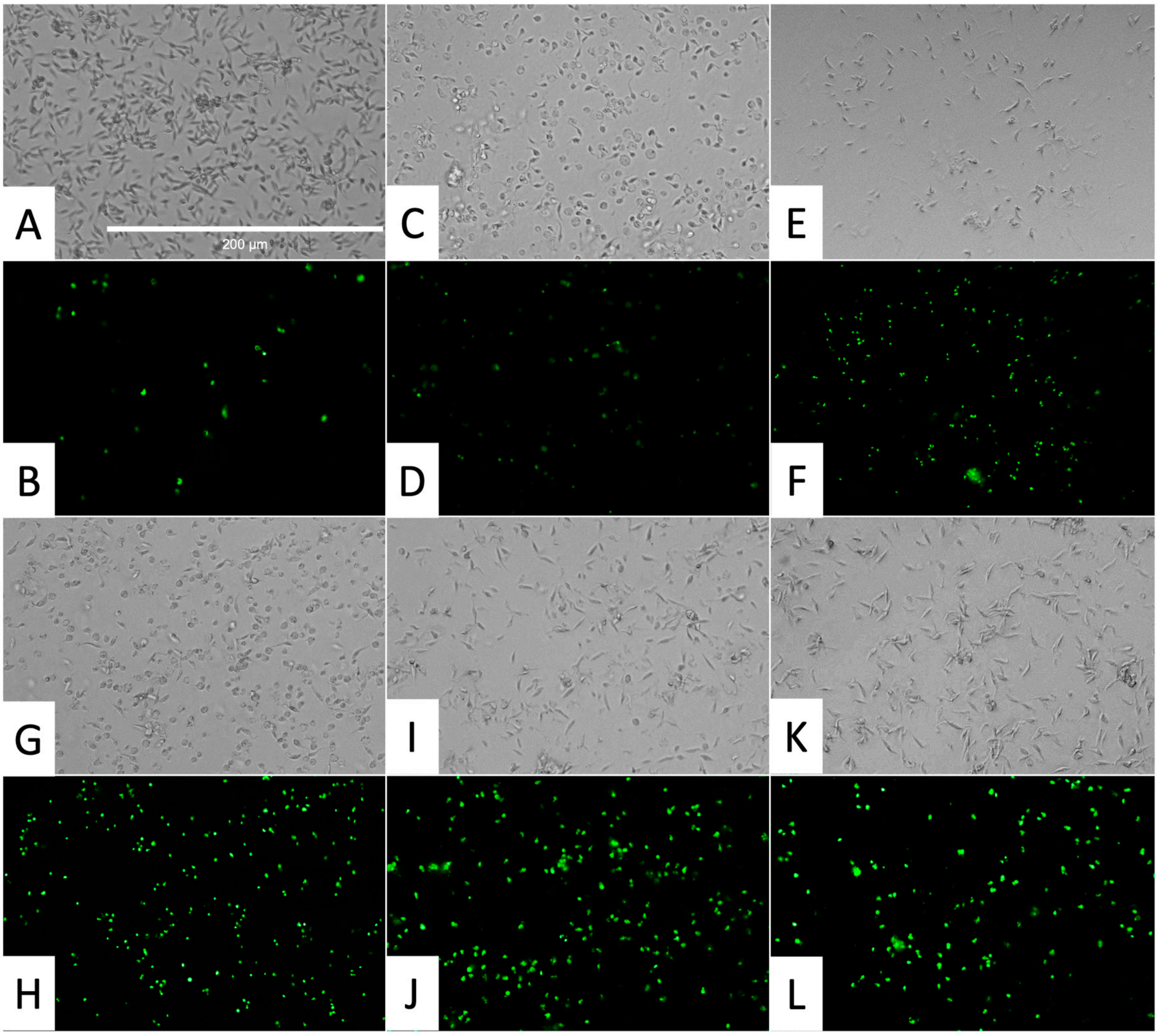

Figure S2. Results of detection of plasmatic membrane permeability in epimastigote stage of *T. cruzi* using SYTOX® Green staining. Images were captured using an EVOS FL Cell Imaging System (40×). Benznidazole was used as reference treatment and triton 0.1% was used as positive control. A: Untreated parasites in visible channel; B: Untreated parasites in GFP channel; C: Parasites treated with benznidazole in visible channel; D: Parasites treated with benznidazole in GFP channel; E: Parasites treated with triton 0.1% in visible channel; F: Parasites treated with triton 0.1% in GFP channel; G: Parasites treated with B in visible channel; H: Parasites treated with B in GFP channel; I: Parasites treated with C in visible channel; J: Parasites treated with C in GFP channel; K: Parasites treated with G in visible channel; L: Parasites treated with G in GFP channel. Scale-bar: 200 µm.

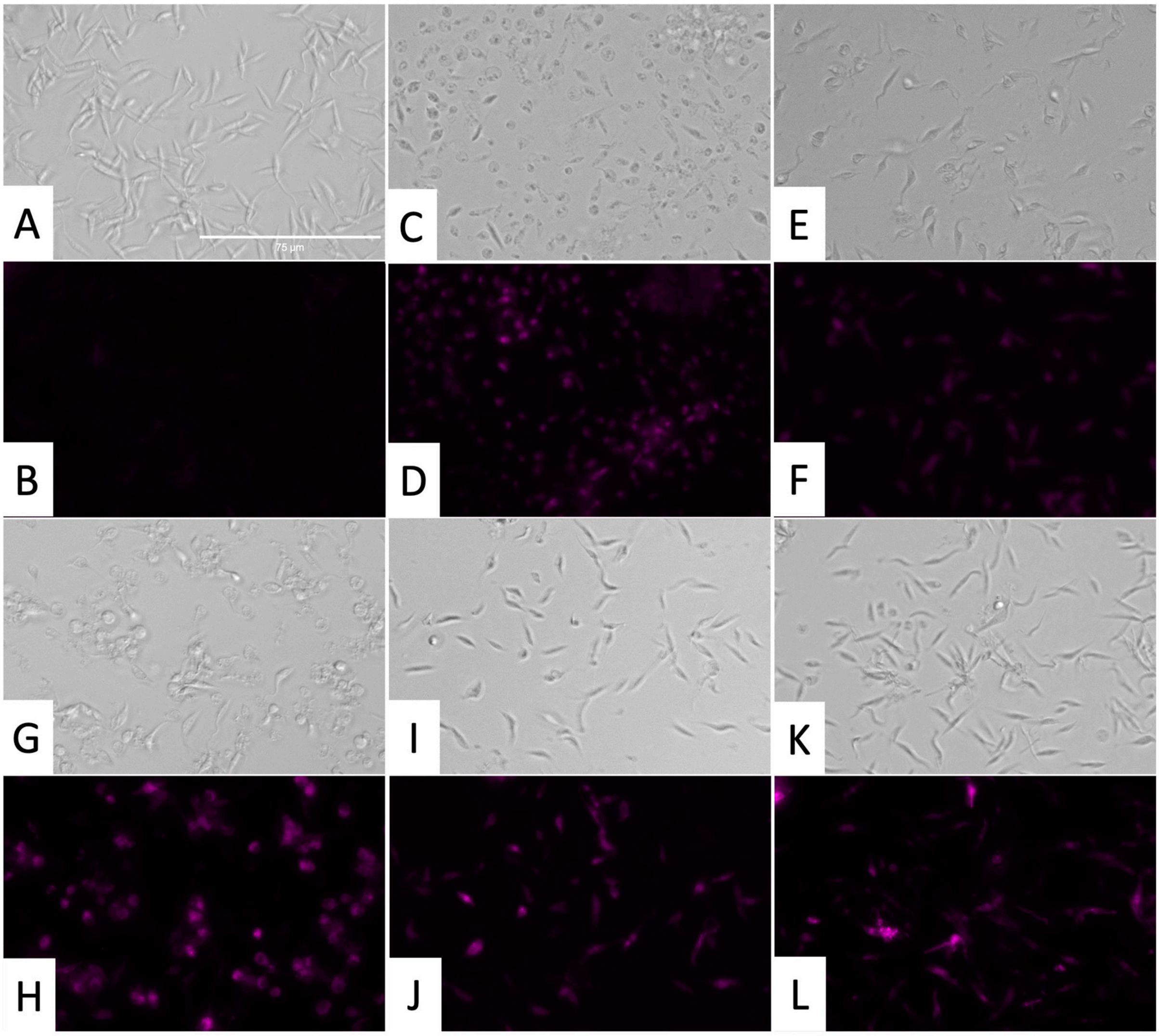

Figure S3. Results of detection of reactive oxygen species in epimastigote forms of *T. cruzi* using CellROX® Deep Red staining. Images were captured using an EVOS FL Cell Imaging System (40×). Benznidazole was used as reference treatment and hydrogen peroxide 600 mM (H_2O_2) was used as positive control. A: Untreated parasites in visible channel; B: Untreated parasites in Cy5 channel; C: Parasites treated with H_2O_2 in visible channel; D: Parasites treated with H_2O_2 in Cy5 channel; E: Parasites treated with benznidazole in visible channel; F: Parasites treated with benznidazole in Cy5 channel; G: Parasites treated with B in visible channel; H: Parasites treated with B in Cy5 channel; I: Parasites treated with C in visible channel; J: Parasites treated with C in Cy5 channel; K: Parasites treated with G in visible channel; L: Parasites treated with G in Cy5 channel. Scale-bar: 75 µm.
